# 10 years of BiTE immunotherapy: an overview with a focus on pancreatic cancer

**DOI:** 10.3389/fonc.2024.1429330

**Published:** 2024-12-20

**Authors:** Solange R. Paredes-Moscosso, Amit C. Nathwani

**Affiliations:** ^1^ Centro de Genética y Biología Molecular, Instituto de Investigación, Facultad de Medicina Humana, Universidad de San Martín de Porres, Lima, Peru; ^2^ Facultad de Ciencias de la Salud, Universidad Peruana de Ciencias Aplicadas, Lima, Peru; ^3^ Research Department of Haematology, UCL Cancer Institute, University College London, London, United Kingdom; ^4^ Katharine Dormandy Haemophilia and Thrombosis Unit, Royal Free London National Health Service (NHS) Foundation Trust, London, United Kingdom

**Keywords:** bispecific antibodies, PDAC, blinatumomab, solitomab, EpCAM BiTE, tarlatamab, ROR1 BiTE, T-cell engagers

## Abstract

Various therapeutic strategies have been developed to treat Pancreatic Cancer (PaCa). Unfortunately, most efforts have proved unfruitful, as the poor prognosis observed in this disease has only attained little improvement in the past 40 years. Recently, deeper understanding of the immune system and its interaction with malignant tumors have allowed significant advances in immunotherapy. Consistent with this, some of the most promising approaches are those that involve T-cell redirection to the tumor site, such as bispecific T-cell engagers (BiTEs). These recombinant antibodies bridge cytotoxic T-cells to tumor cells, inducing target cell-dependent polyclonal T-cell activation/proliferation, which in turn results in elimination of bound tumor cells. Blinatumomab, an anti-CD19 BiTE, received FDA approval in 2014 for Precursor B-cell Acute Lymphoblastic Leukemia. In the past decade, it has demonstrated impressive clinical benefit in patients with B-cell leukemias; and other T-cell engagers have been FDA-approved for hematological malignancies and other diseases, yet limited effect has been observed with other BiTEs against solid cancers, including PaCa. Nevertheless, on May 2024, Tarlatamab, an anti-DLL3 BiTE was approved by the FDA for extensive small cell lung cancer, becoming the first BiTE for solid tumors. In this review, the generation of BiTEs, therapeutic features, manufacturing issues as well as the remaining challenges and novel strategies of BiTE therapy in the context of PaCa, including the lessons we can learn from the use of BiTEs on other types of cancer will be explored.

## 1 Introduction

It is now widely accepted that immune escape is a tumor feature that constitutes a hallmark of cancer (1, 2). This relationship between immunity and cancer has been studied for over a century since William Coley reported successful treatment of inoperable sarcoma by mixed bacterial toxins (*S. erysipelas* and *B. prodigious*) (3). In 1909, Paul Ehrlich postulated for the first time the concept of ‘tumor surveillance’, which proposed that cancer cells were distinguishable from healthy cells and could therefore be eliminated by the immune system before clinical detection (4). A few decades later, Lewis Thomas and Frank MacFarlane Burnet provided the first experimental evidence that supported the ‘cancer immunosurveillance’ hypothesis using homograft rejection studies (5, 6)

After these encouraging results, almost 30 years of contradicting studies stagnated the field and raised skepticism amongst immunologists and clinicians. This changed since the 1990s, as a greater understanding of cellular immunity and the generation of better animal models were increasingly available. New reports revealed that, apart from immunosurveillance, the immune system promoted the formation of primary tumors with reduced immunogenicity, able to escape immune recognition and destruction (7). This prompted the ‘cancer immunoediting’ hypothesis, a dynamic process composed of 3 phases: elimination, equilibrium and escape. For relevant reviews, please refer to: Dunn et al., 2004 (8) and O’Donnell et al., 2018 (9).

More recently, treatments based on cancer immunology have achieved important clinical outcomes (10). The knowledge that both: i) expression of the cytotoxic T lymphocyte associated antigen 4 (CTLA-4), an inhibitory receptor on regulatory T-cells (Tregs) is a key regulator of T-cell activation (11); and ii) overexpression of the programmed death-ligand 1 (PD-L1), or its receptor on T-cells (PD-1), promotes dysfunction of tumor-infiltrating T-cells (12) has led to the targeting of these molecules using monoclonal antibodies. Certainly, the latter have proved to be an effective therapeutic option against a range of solid tumors, including melanoma, non-small cell lung cancer and renal carcinoma (13–15).

In this context, cancer immunotherapy can be defined as the approach to treating cancer by generating or augmenting an immune response against malignant tumors (16), whereby both the innate and adaptive arms of immunity can target tumor cells. Importantly, this approach has broad potential and offers the possibility of achieving durable and robust responses across a diverse spectrum of malignancies (17–19)

Accordingly, significant advances in the use of therapeutic vaccines, adoptive cellular therapy, monoclonal antibodies and its derivatives have boosted the field (20). Amongst the latter, it was the clinical benefit triggered by immunomodulating antibodies capable of activating endogenous T-cells by blocking immune checkpoints in solid malignancies that led cancer immunotherapy to be named “2013’s Breakthrough of the Year” by *Science* (21). Similarly, bispecific antibodies are another promising approach to fight cancer cells, particularly those with the ability to redirect effector immune cells such as Bispecific T-cell engagers (BiTEs). This type of molecules can recognize two different antigens simultaneously, which allows T-cells to be in close proximity to malignant cells resulting in their elimination (14).

In the following sections, the context in which BiTEs were generated, their structure, therapeutic features, and manufacturing challenges will be discussed. Next, the current status of BiTE therapy in pancreatic cancer will be explored, including some preclinical data reported in the literature as well as the remaining challenges that need to be approached and the novel strategies that are being developed. In addition, BiTEs used in other types of cancer, currently present in the clinic, will be briefly mentioned. Finally, some concluding remarks on BiTE therapy in pancreatic cancer will also be provided.

## 2 BiTEs

### 2.1 Antibodies as bispecific molecules

As reviewed by Strebhardt and Ullrich, Paul Ehrlich first proposed the “magic bullet” hypothesis over a century ago; yet it was the development of the hybridoma technology by Kohler & Milstein (22) that allowed the generation of monoclonal antibodies (MAbs) to become feasible (23).

Antibodies are globular proteins, called immunoglobulins, produced by B cells. They are the most diverse proteins found in nature as they are deployed by the immune system to target foreign molecules overexpressed on the surface of affected cells. Cell targeting is elicited with high affinity and specificity rendering them susceptible to immune destruction (24). Based on their structure, these immune molecules are classified into 5 different isotypes: IgA, IgG, IgM, IgD and IgE. Of these, the IgG isotype outstands due to its potent effector functions (25).

**Figure 1:**
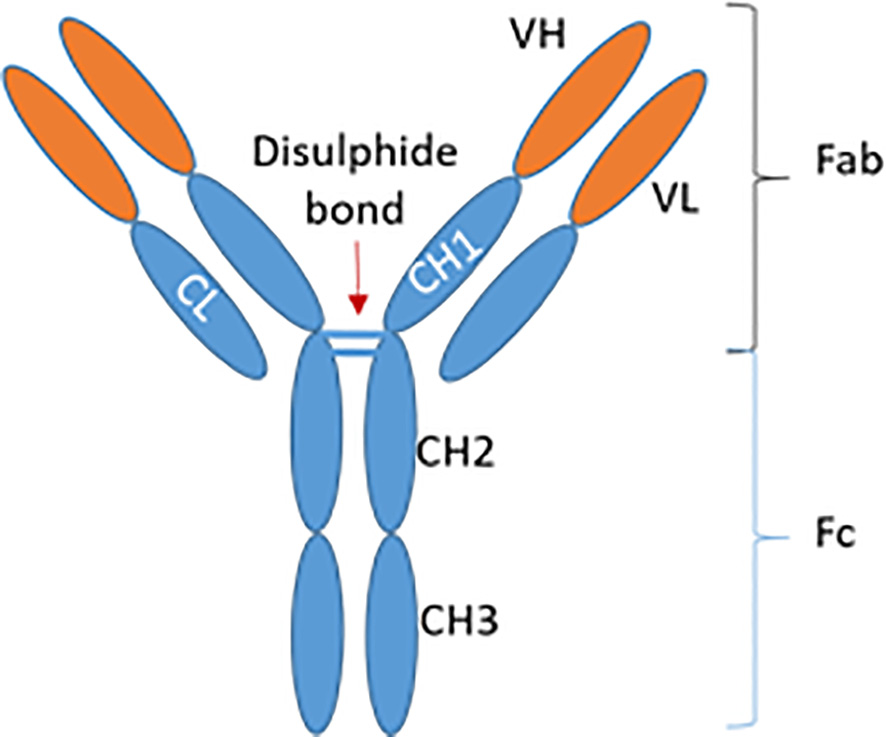
Structure of an IgG antibody. IgG, Immunoglobulin G; VH, Variable heavy chain; VL, Variable light chain; CL, Constant light chain; CH, Constant heavy chain; Fab, Fragment antigen-binding; Fc, Fragment crystallizable region. Adapted from Krishnamurthy & Jimeno, 2018 (36).

Immunoglobulin G consists of two light and two heavy chains, which in turn are composed of variable and constant regions. Each half of the IgG is connected by disulphide bonds, and together possess a total molecular weight of 146-160kDa. Antigen-binding sites are formed by hypervariable regions of two heavy (V_H_, 55-70kDa) and two light (V_L_, 25kDa) chains. Thus, antibodies are normally monospecific and bivalent as they contain two identical antigen-binding sites. Notably, the greatest variability amongst IgG antibodies lies in the amino acid sequence contained within the hypervariable regions, namely the complementarity-determining regions (CDRs). The latter determines therefore the specificity of the antibody to its cognate antigen (See **Figure 1**).

Monoclonal antibodies are secreted by identical immune cells, clones of a single parent cell, and can bind the exact same epitope. In the past two decades, the use of MAbs has become an established strategy for the treatment of both hematological and solid malignancies (26), either as monotherapy or in combination with chemotherapy, small-molecule inhibitors and other antibodies (27). Certainly, naked antibodies have improved overall response rate, complete remission rates, and progression-free as well as overall survival in multiple cancers including breast cancer, colon cancer, lymphomas, amongst others (28).

Based on their target, antibodies used in cancer treatment can be classified into two categories:

- direct targeting MAbs; comprised of conventional antibodies that target tumor cells by direct binding to either lineage-specific antigens (such as CD20 or CD52), tumor neoantigens (e.g. glycans) or oncogenic biomarkers [e.g. epidermal growth factor receptor, EGFR (29)]; and
- immunomodulatory MAbs; which do not engage tumor cells directly but target receptors on immune cells in an attempt to overcome immunosuppression from the tumor microenvironment (30).

Moreover, researchers in the field of cancer immunotherapy are also focusing on the activation of the immune system, particularly CD8^+^ T-cells (31, 32).

Bispecific antibodies are based on monoclonal antibodies but with the ability to recognize and bind to two different targets simultaneously. The concept of such antibodies stemmed from the knowledge that there are multiple factors contributing to disease; therefore, the simultaneous blockade of different targets (epitopes) could result in better efficacy (33). Accordingly, in 2014, the journal *Nature Reviews Drug Discovery* called them “next generation antibodies”. To date, a decade later, 10 bispecific molecules have been approved for therapeutic use (34). See **Table 1**:

**Table 1:** FDA-approved bispecific antibodies.

Trade Name	Target	Active Ingredient	Year approved	Indication
Blincyto	CD19xCD3	Blinatumomab	2014	To treat Philadelphia chromosome-negative relapsed or refractory B cell precursor acute lymphoblastic leukemia
Hemlibra	FIXa x FX	Emicizumab-kxwh	2017	To prevent or reduce the frequency of bleeding episodes in hemophilia A with factor VIII inhibitors
Rybrevant	EGFRxMET receptor	Amivantamab-vmjw	2021	To treat locally advanced or metastatic non-small cell lung cancer with certain mutations
Kimmtrak	HLA-A2xCD3	Tebentafusp-tebn	2022	To treat a form of unresectable or metastatic uveal melanoma
Vabysmo	VEGFxAng2	Faricimab-svoa	2022	To treat neovascular (wet) age-related macular degenerated and diabetic macular edema
Tecvayli	BCMAxCD3	Teclistamab-cqyv	2022	To treat relapsed or refractory multiple myeloma
Lunsumio	CD20xCD3	Mosunetuzumab-axgb	2022	To treat relapsed or refractory follicular lymphoma
Epkinly	CD20xCD3	Epcoritamab-bysp	2023	To treat relapsed or refractory diffuse large B-cell lymphoma
Columvi	CD20XCD3	Glofitamab-gxbm	2023	To treat relapsed or refractory diffuse large B-cell lymphoma or large B-cell lymphoma
Talvey	GPRC5DxCD3	Talquetamab-tgvs	2023	To treat adult patients with relapsed or refractory multiple myeloma (RRMM) who have received at least 4 prior lines of therapy, including a proteasome inhibitor, an immunomodulatory agent, and an anti-CD38 monoclonal antibody
Imdelltra	DLL3xCD3	Tarlatamab-dlle	2024	To treat adult patients with extensive-stage small cell lung cancer (ES-SCLC) with disease progression on or after platinum-based chemotherapy

Furthermore, the number of novel antibody candidates entering clinical trials have gone from 63 during the early 2010s to over 140 by 2020 (35).

Based on format, bispecific antibodies can be subdivided into 2 groups: i) Ig-like, accounting for those that possess an Fc region, and ii) Bispecific fragment molecules, referring to those that lack an Fc portion. Additionally, and as reviewed by Krishnamurthy & Jimeno, there are four areas in which developing bispecific antibodies is pivotal: 1) inhibition of two cell surface receptors, 2) blocking of two ligands, 3) cross-linking of two receptors, and 4) recruitment of immune effector cells such as T-cells (36).

Although MAbs have revolutionized cancer therapy, they are not able to cure most cancers and are usually administered in combination with other agents. It has been proposed that this might be due in part to the fact that T-cells do not take an active role in antibody-mediated tumor destruction. MAbs either prevent the binding of growth factors to the receptors or block the inhibitory signals on immune cells (37).

In this context, the advancement of genetic engineering has allowed for greater flexibility in the design of bispecific fragments able to bind to cancer cells and activate the immune system simultaneously. These molecules can now be found in different formats, such as Tandem antibodies (TandAbas), Immune-cell-mobilizing monoclonal TCRs against cancer (ImmTACS), Diabodies, dual-affinity-retargeting format (DART), Dual-action Fab (DAF), Bispecific T-cell engagers (BiTEs), amongst others (14). Of these, one of the most used formats that has reached FDA approval -in the context of cancer- is the BiTE antibody construct as it has emerged as a particularly promising option in terms of safety, cost and relative ease of production (34, 38).

### 2.2 Structure

**Figure 2:**
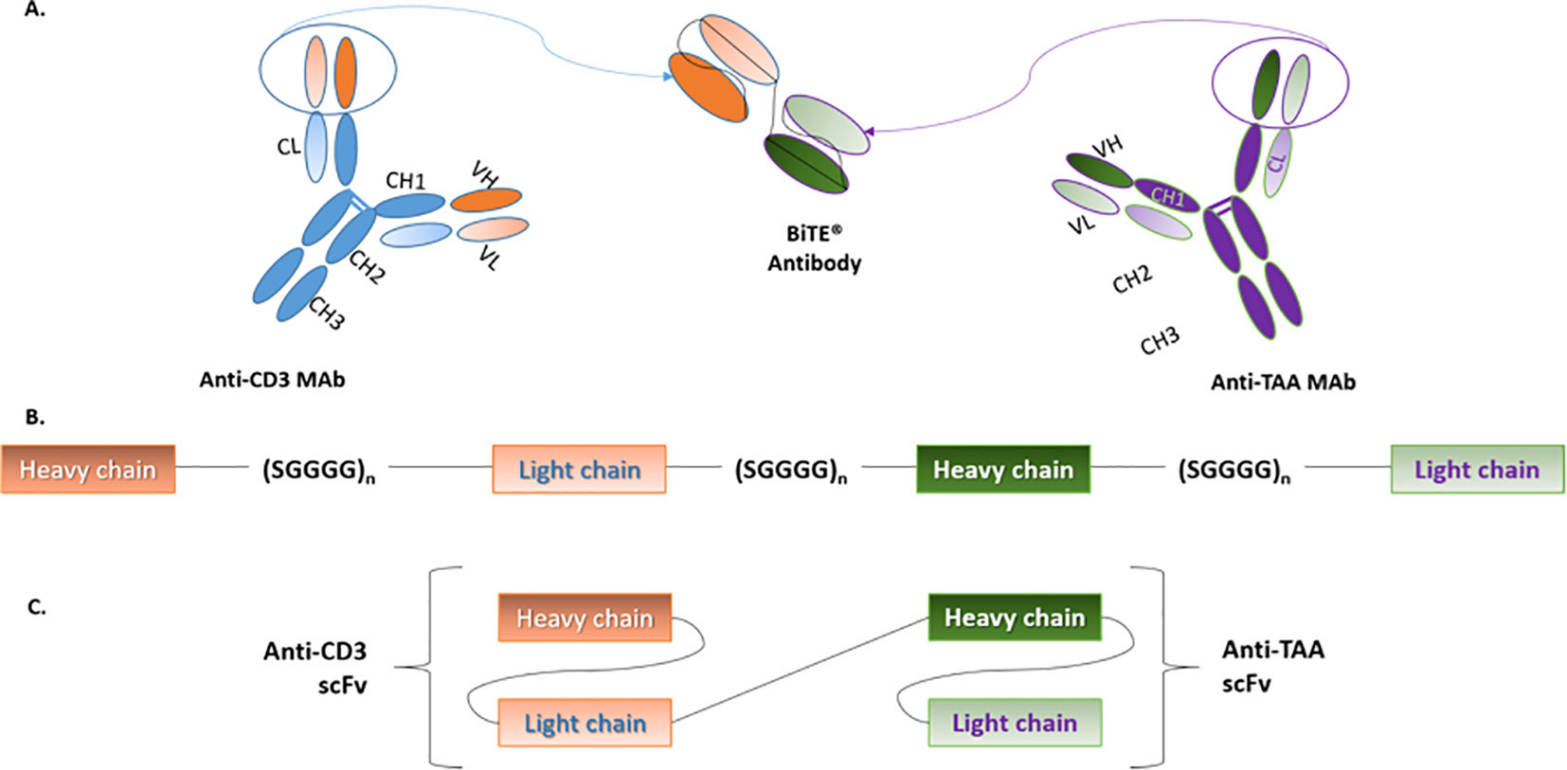
BiTE structure. **(A)** BiTEs are generated by genetically linking the scFvs of two MAbs; one for CD3 on T-cells and the other for TAAs on cancer cells. **(B)** The variable heavy and light chains of each scFv are connected by short serine-glycine linkers. Similarly, both scFvs are linked by a serine-glycine sequence, which results in the formation of a single polypeptide that contains both scFvs. **(C)** Although, in structure, BiTEs are a type of tandem scFv, their conformation allows them to effectively bridge T-cells and cancer cells. BiTE, Bispecific T-cell engager; CD, Cluster of differentiation; Mab, Monoclonal antibody; TAA, Tumor-associated antigen. Adapted from Huehls et al., (43) and Stieglmaier et al., (40).

A BiTE antibody is a 55-60kDa recombinant, non-glycosylated protein. It is approximately 11nm in length and it is composed of scFv portions of two different MAbs. Each scFv possesses a unique antigen specificity, covalently connected via a glycine-serine 5-amino acid non-immunogenic linker (SGGGG repeats) (38). The latter sequence allows the linker to be long and flexible enough as to permit both the variable heavy (V_H_) and variable light (V_L_) chains to associate in a normal conformation. Of note, the linker that connects both scFvs together also contains a SGGGG amino acid sequence as it will determine the flexibility with which the BiTE will target both cell types (39, 40) (See **Figure 2**).

### 2.3 Mechanism of action: T-cell activation

**Figure 3:**
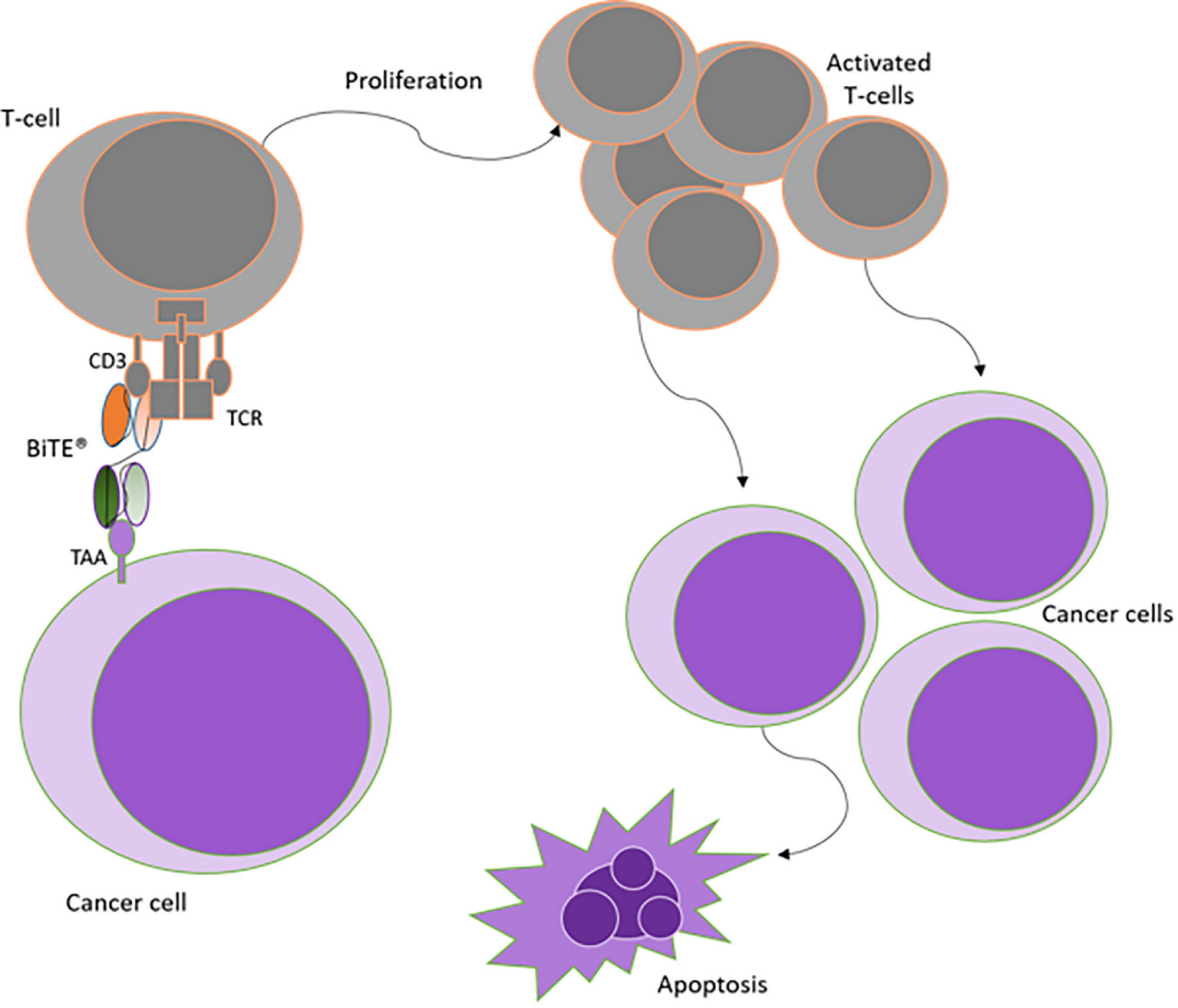
Mechanism of action of BiTE antibody constructs. Simultaneous binding of BiTEs to both T-cells and cancer cells is required to activate the immune cells. Upon BiTE binding, an immunological synapse is formed, thereby achieving T-cell activation independent of TCR specificity or costimulation. Activation is followed by T-cell proliferation, which amplifies the immune response against cancer cells leading to apoptosis of target cells. Adapted from Stieglmaier et al., (40).

In the BiTE format, one arm binds to the CD3ϵ subunit of the T-cell receptor (TCR) complex, whilst the second scFv targets a specific tumor-associated antigen (TAA) expressed primarily on cancer cells. BiTEs can hence redirect and bring endogenous polyclonal T-cells to sites of tumor, leading to the formation of immunological synapses. This results in the activation and proliferation of T-cells. Cytotoxicity is then elicited by the release of perforin and granzymes from granules in the cytotoxic T-cell. This in turn prompts a calcium-dependent proteolytic activation of intracellular caspases which induces lysis of the malignant cell, independent of MHC/TCR interactions or the presence of costimulatory molecules (38) (See **Figure 3**).

Interestingly, cytotoxic activity in the absence of costimulation seems to be unique to BiTE technology, as other types of bispecific antibodies have reportedly required costimulation in order to exert their cytotoxic effect (41, 42). Up to date, there is no clear explanation of this phenomenon. Yet, it has been hypothesized that many TCR molecules group together within the BiTE-induced immunological synapse, which might be enough to trigger signaling (43). Alternatively, Dreier et al. have shown that BiTEs preferentially recruit cytotoxic memory T-cells, which require less stimulation to be fully activated (44). Also, a recent study by Gonnermann et al., showed that bispecific antibodies targeting γδ T-cells enhanced their cytotoxic effect against pancreatic cancer cells and overcame the immunosuppressive function of galectin-3 (45). Further investigation is needed to understand the complexity of the BiTE-induced immunological synapse and determine the precise phenotype of the effector cells BiTE molecules recruit.

Importantly, it has been demonstrated that the simultaneous binding to both CD3 and TAA is required to elicit cell death (46), as single-sided binding to T-cells does not induce T-cell activation, anergy or cytokine release (47). Although this feature contributes to the safety profile of BiTEs, it also emphasizes the importance of having T-cells around the bispecific antibody molecules. Hence, it might explain in part why BiTEs are not as effective in solid tumors compared with liquid cancers, as the availability of T-cells is significantly reduced within the tumor microenvironment (TME) of solid cancers than in leukemias.

BiTE binding to both TAA and CD3 results in the activation and polyclonal expansion of CD3^+^ immune cells, including the upregulation of activation markers CD25 and CD69. Furthermore, video microscopy experiments have shown that T-cells exposed to Blinatumomab, the first-in-class clinically approved CD19xCD3 BiTE, remain activated and continue to produce and store perforin and granzymes in order to serially attack additional CD19^+^ cells (48, 49). Of note, inflammatory cytokines are also released, and T-cell proliferation is further promoted after administration of Blinatumomab (40, 50).

### 2.4 Pharmacological features

Unlike MAbs or bispecific antibodies in full IgG formats, BiTEs may have a better chance to penetrate deep into solid tumors due to their small size (55-60kDa). At such privileged location, they have the potential to re-activate local tumor-infiltrating lymphocytes (TILs) or even co-opt Tregs (51). Furthermore, the short half-life of these bispecific binders offers a reduced clinical risk in the scenario of adverse events caused by BiTE therapy.

Compared to T-cell immunotherapies, such as chimeric antigen receptor (CAR) immune cells, BiTEs can exploit the resident polyclonal T-cell population and do not rely on a particular subset. Data from several studies indicate that, in addition to CD8^+^ cytotoxic T-cells, CD4^+^ helper T-cells are also able to upregulate production of perforin and granzyme B upon BiTE-mediated binding to target cells (38, 52, 53). Furthermore, this type of therapy does not require *ex vivo* manipulation.

In terms of clinical benefit, a long-term survival analysis of patients with minimal residual disease after chemotherapy showed 61% hematologic relapse-free survival at a median of 33 months after Blinatumomab (50). Despite these encouraging results, better strategies for managing concomitant major adverse events such as cytokine release syndrome (CRS), seen in both CD19 BiTE therapy and CD19 CARs, are required.

As reviewed by Sedykh et al., some of the most common side effects during treatment with Blinatumomab are: lymphopenia, leukopenia, thrombocytopenia, hepatotoxicity, chills, fever, pyrexia, nausea and vomiting (37). Nevertheless, major neurological and psychiatric side effects are completely reversible once the treatment is completed (54).

In terms of the mechanisms of resistance in BiTE therapy, loss of TAA expression has been observed. Acute lymphoblastic leukemia (ALL) patients treated with Blinatumomab in a Phase II trial, relapsed with CD19-negative B-cell ALL disease (55, 56). More recently, adaptive resistance to BiTEs was described when Kohnke et al. reported for the first-time increased PD-L1 positivity in a 32-year-old male patient with refractory B-precursor ALL resistant to Blinatumomab (57). Also, high frequency of Tregs (defined with a cutoff of 8.525%) in B-precursor ALL patients had a 100% failure rate to Blinatumomab (58). Nevertheless, researchers suggested that therapeutic removal of Tregs by the use of Cyclophosphamide and Fludarabine (as per CAR therapy) could convert Blinatumomab non-responders to responders.

Due to the lack of an Fc region, BiTE antibodies exhibit a short half-life in serum, which presents both benefits and challenges to their use in the clinic. BiTE clearance from human serum has been estimated to last approximately 1.25h (59). This calculation may not account for the BiTE molecules already bound to target cells, underestimating the extended effect they might have in the immune system. Yet, the short half-life of these bispecific antibodies is problematic, as rapid disappearance from the serum has to be counteracted by intermittent infusion of the therapy. Infusion pumps are therefore employed for BiTE administration (60); however, their use can be restricted by the severity of the patient’s clinical situation.

Nonetheless, a short serum half-life can also be beneficial as it allows for controlled dose-escalation, rapid readjustment or withdrawal of BiTEs in case of adverse events, thereby enhancing the safety profile of BiTEs in the clinic. Consistently, major adverse events such as cerebellar effect and seizures reported in Phase I and II clinical studies after Blinatumomab treatment were completely reversible (43, 60).

In **Table 2**, a brief summary of the benefits and disadvantages of the use of BiTEs in the clinic is provided.

**Table 2:** Summary table of the benefits and disadvantages of BiTE therapy.

Benefits	Disadvantages
Flexibility: Small fusion linker allows the BiTE antibody to rotate freely, facilitating optimal T-cell and target cell interactions.	Short serum half-life: In humans, BiTEs have an approximate half-life of less than 2h, which requires continuous administration of the drug through a pump.
Versatility: Cytotoxic effect is independent of MHC interactions and costimulatory molecules.	Continuous infusion: This form of administration might be challenging for some patients, depending on the severity of their clinical situation.
Modular design: Due to its format, the BiTE platform can be easily used with a variety of TAAs across a range of cancer types.	Manufacturing issues: Lack of stability (scFv aggregation), purification difficulties, low expression titres and poor solubility.
Potent effect: Cytotoxicity can be achieved even at very low concentrations (1ng/ml in *in vitro* models).	Therapy resistance: Loss of targeted TAA expression (Relapse), T-cell exhaustion.
Low risk of non-specific toxicity: Simultaneous binding to TAA and CD3 is required for cytotoxicity.	Adverse events: Cytokine release syndrome and neurotoxicity when administered in high doses.
Safety: Due to its short serum half-life, BiTE administration can be increased in a stepwise manner, quickly readjusted or even withdrawn in case of adverse events.	
Immunoactivation-cytokines: BiTE-activated T-cells secrete pro-inflammatory cytokines: IFNg, TNFa, IL2, IL4, IL6 and IL10.	
Immunoactivation-immune cells: Apart from CD8+, evidence suggests BiTEs can also bind CD4+ helper T-cells and co-opt Tregs, leading to an expanded anti-tumour T-cell response.	
Relative ease of production: No *ex vivo* production required and much cheaper than other T-cell immunotherapies: “off the shelf” products.	

### 2.5 Manufacturing

The first attempts to produce bispecific antibodies pertained those in full IgG formats. To achieve this in the lab, technical procedures encompassed chemical conjugation of two different purified monoclonal antibodies by oxidative recombination (61). Alternatively, quadroma technology, which is based on the somatic fusion of two hybridoma cell lines, was also used. Unfortunately, the high heterogeneity of the products made purification significantly challenging (62).

Advances in genetic engineering have permitted the development of IgG-deprived antibody derivatives, such as bispecific T-cell engagers; for which production and purification have proved less convoluted (38). Although some of them were first produced using *Escherichia coli* systems, bispecific antibodies such as BiTEs are mainly produced using mammalian cell lines, specifically Chinese Hamster Ovary (CHO) cells. The latter secrete the bispecific binders into the cell culture medium in a non-glycosylated form for subsequent purification (38). Certainly, a major advantage of using eukaryotic systems is that proteins are generated with fewer folding errors and greater efficiency, which permits posterior scaling up for clinical translation.

Nevertheless, due to their smaller size and lack of Fc region, BiTEs tend to be unstable molecules with a certain predisposition to form aggregates. Notably, most BiTEs including Blinatumomab contain a hexahistidine tag at the C-terminus to allow efficient capture and purification using metal affinity chromatography on a nickel-nitrilotriacetic acid (Ni-NTA) column (63).

Importantly, and according to Zhang et al., bispecific antibodies need to meet some ‘developability’ standards for clinical translation. Such criteria consist of stability, low tendency to form aggregates or to accumulate chemical deviations, and the ability to be formulated at high concentrations without viscosity issues (64). In line with this, Blinatumomab production was further optimized by introducing downstream processes such as gel filtration, by which unwanted multimers are removed (44).

## 3 BiTEs in pancreatic cancer

### 3.1 Overview of pancreatic cancer

**Figure 4:**
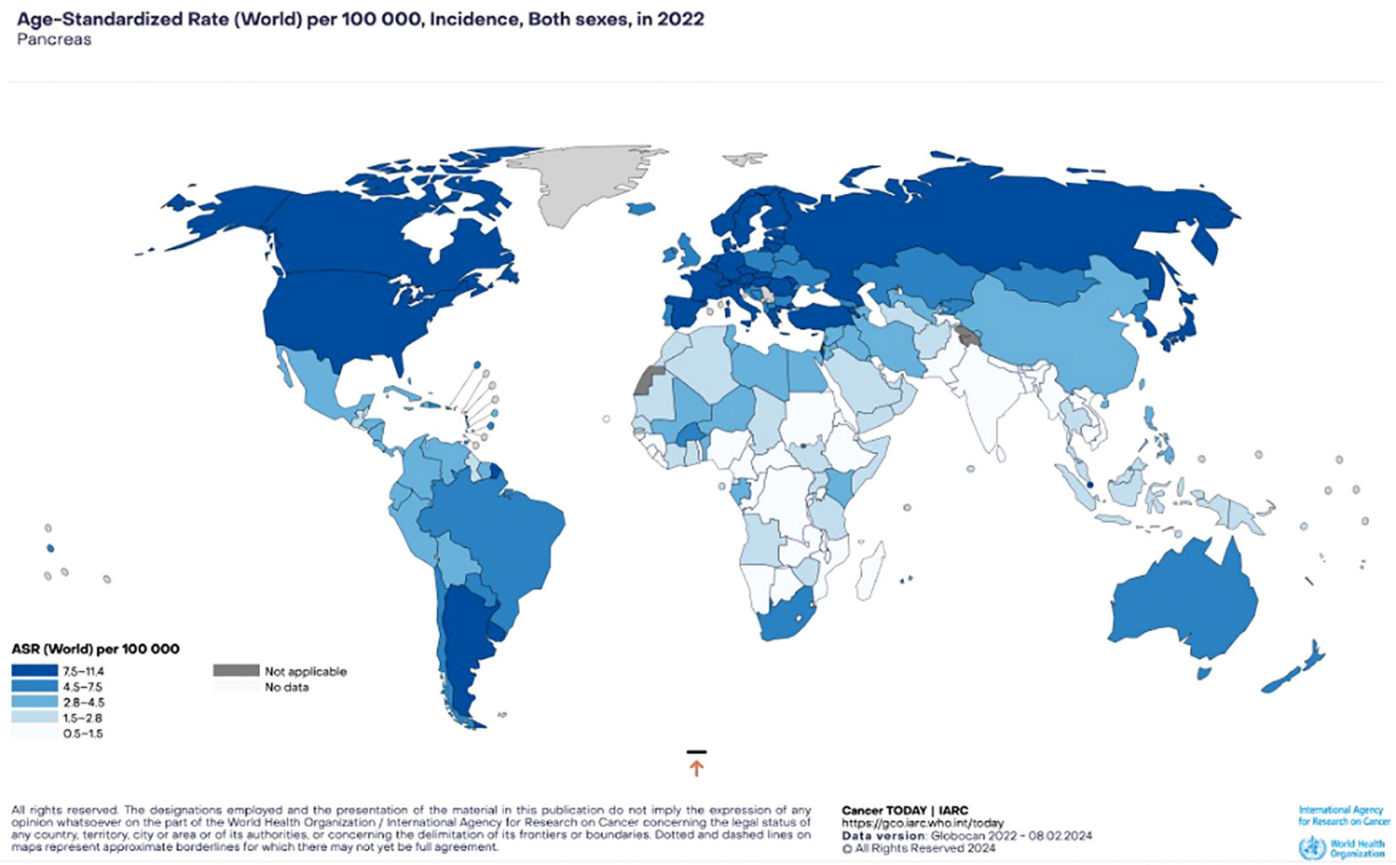
Pancreatic cancer incidence. Estimated age-standardized incidence rates (World) in 2022, both sexes, all ages (GLOBOCAN 2022).

According to Siegel et al., PaCa is projected to be the third leading cause of cancer-related death in the US up to 2020 and the 7th worldwide (65, 66). In terms of incidence, Western countries show the highest rates with approximately 6 new cases per 100 000 habitants, followed closely by Asia and Latin America; whilst the African continent reports an average of less than 3 new cases per 100 000 habitants (67, 68) (**Figure 4**).

In the past 40 years, only little improvement has been achieved regarding the extremely poor prognosis observed in this malignancy (69). Even with currently available therapies, a median survival of less than 6 months and overall 5-year survival rates of 1-5% are obtained (70). For a more detailed yet concise description of PaCa biology, tumor microenvironment (TME) and current therapies, including BiTEs, please refer to (19, 71).

A major hurdle impeding early detection of this disease is the absence of symptoms and lack of specific diagnostic markers (72). The majority of patients (80-90%) is therefore diagnosed when local metastasis has already occurred. Once detected, the standard therapy for patients with metastatic disease is Gemcitabine-based chemotherapy. However, the long-term efficacy and prognosis of this approach varies widely and is usually unsatisfactory for most PaCa patients (73, 74). Notably, patients eligible to benefit from surgical resection -currently the only curative option- are those diagnosed at early stages of the disease. Said patients however do still have poor prognosis and more than 80% of them relapse within 2 years after surgery (75).

As proposed by Warshaw and Fernandez-del Castillo, pancreatic cancer can be classified into two categories depending on the cell type the tumor originates from: exocrine and endocrine tumors (76). Of these, Pancreatic Ductal Adenocarcinoma (PDAC) -an exocrine tumor- is a major histological subtype and represents more than 90% of all PaCas (68, 77, 78). PDACs are solid and firm tumors with a highly metastatic behavior, which results in rapid tumor spreading to lymph nodes and liver.

The high mortality seen in pancreatic cancer is often associated to the highly dense fibrotic tissue as it effectively acts as a physical barrier to drug delivery. This is further accentuated by the fact that said dense fibrotic tissue constitutes 90% of the tumor volume. Moreover, PDAC shows minimal response to chemotherapy, probably due to the presence of cancer stem cell-like cells (CSCs) within the tumor (70, 77). Consistently, CSCs have been associated to tumorigenesis, metastasis, chemotherapy resistance and poor clinical outcome. Furthermore, accumulating evidence suggest that these cells are highly resistant to chemotherapy and their presence may therefore account for the rapid and virtually universal relapse observed in PDAC (77, 79).

As a whole, the complex genetic heterogeneity, highly dense tissue composition of PDAC, invasive nature of the tumor and the presence of CSCs make PaCa, in general, and PDAC in particular a challenging disease to treat. Strategies that combine bypassing the immunosuppressive TME and CSC targeting will prove instrumental to PDAC therapy.

### 3.2 BiTE therapy in pancreatic cancer: reported examples

As briefly mentioned earlier, Blinatumomab is the first BiTE that received accelerated FDA approval in 2014 for the treatment of ALL. It targets CD19, an antigen expressed on the cell surface of both malignant and healthy B cells (80). Due to its modular design, BiTE technology can be easily engineered to allow targeting of a variety of antigens expressed on different cell types, including PaCa cells.

MT-110 is an anti-EpCAM x anti-CD3 BiTE. Epithelial cell adhesion molecule (EpCAM) is commonly upregulated and functionally altered in pancreatic cancer cells, including CSCs (81, 82). Whilst certain healthy epithelial tissue express EpCAM, it is mostly sequestered within intercellular boundaries but becomes accessible on the surface of disintegrated cancer cells. Therefore, when EpCAM-targeting BiTEs (also known as Solitomab) were tested on primary human PaCa CSCs *in vitro* and *in vivo*, elimination of these cells was achieved in a time- and dose-dependent manner without significant toxicity (83). Solitomab has since been tested in a Phase I clinical trial in patients with different types of solid tumors; unfortunately, pancreatic cancer was not investigated (84).

More recently, the effect of BiTEs targeting carcinoembryonic antigen (CEA) was studied in a Phase I clinical trial on patients with gastrointestinal adenocarcinomas, including pancreatic cancer (71, 85). Unfortunately, no objective responses were observed, and 48.7% of all patients developed antidrug antibodies. Only 28% of patients had stable disease as best response. This is in contrast with the recent publication of results from a Phase I clinical trial of hypoxia-responsive CEA CAR-T cell therapy in patients with heavily pretreated solid tumor via intraperitoneal or intravenous transfusion. Stable disease was observed in 62.5% of i.p-infused patients vs 58.3% of i.v.-infused participants. Although all patients experienced grade 3-4 hematologic toxicity and 32.5% presented immune-mediated diarrhea and colitis of all grades, promising anti-tumor potential was observed in the i.p. group (86). These results warrant further studies for CEA BiTE and CAR therapy, including the revision of patient recruitment criteria.

At the preclinical level, Gohil et al. developed a BiTE antibody against receptor tyrosine kinase/like orphan receptor 1 (ROR1), a transmembrane antigen overexpressed on malignant cells and CSCs but not on healthy critical tissue. The researchers showed that co-culture of a range of ROR1-expressing pancreatic cancer cell lines with unstimulated T-cells and ROR1 BiTE resulted in specific *in vitro* cytotoxicity even at very low concentrations (0.1ng/ml). When tested *in vivo*, ROR1 BiTE prevented engraftment of pancreatic tumor xenografts in mice and reduced the size of established subcutaneous tumors by at least 3-fold (87).

More recently, the cytotoxic effect that the ROR1 BiTE had on a PDAC cell line-derived CSC model was assessed. Immunocytochemistry data on such tumorsphere model suggested that ROR1 BiTE elicited specific *in vitro* elimination of cells expressing both CSC biomarkers and ROR1 (19). Further preclinical and clinical studies are needed in order to establish the therapeutic potential that ROR1 BiTE could have on pancreatic cancer either alone or in combination with other treatments. Interestingly, this BiTE is currently known as NVG-111 (Novalgen) (88) and is now being tested in Phase I clinical trials for CLL and Mantle Cell Lymphoma (MCL). Up to November 2023, NVG-111 had been tested in 12 patients who completed a maximum of 6 cycles of treatment. Despite time limited exposure to this anti-ROR1 BiTE, the restricted mean survival time for duration of response (DoR) was 13.6 months (SEM 3.07). The median was not yet calculable as response was still ongoing in four subjects. The median progression-free survival was 18.7 months (95% CI 2.6 - not calculable) (89). These results provide evidence for targeting ROR1 in the context of hematological malignancies and encourage further studies on solid tumors, such as PDAC.

### 3.3 Remaining challenges: TME, T-cell surveillance, etc.

Malignant tumors are supported and surrounded by a highly complex and heterogeneous ecosystem called the tumor microenvironment (TME). The latter is composed of cancer cells, stromal fibroblasts, endothelial cells and various types of immune cells that regulate tumor growth and invasion. For a broad and valuable review on the clinical role of the TME in various types of solid cancers, please refer to Giraldo et al., 2019 (90).

Histology studies in the pancreas have shown strong epithelial wound healing in chronic disease (91). Such stromal response includes fibroblast activation, immune suppression, remodeling of the extracellular matrix as well as trophic signals to promote re-epithelialization (92). Remarkably, it has been hypothesized that the features observed during the wound healing process are the same as those exploited by the TME during PDAC (93).

Furthermore, previous reports indicate that pancreatic stellate cells (PSCs) and other mesenchymal cells are constantly activated by the epithelium through secretion of TGFβ, sonic hedgehog (SHH) and platelet-derived growth factor (PDGF). In line with this, Sherman et al. reported that *in vivo* pharmacological inhibition of PSC activation using PDAC murine models resulted in stromal collapse, smaller tumors, and improved chemotherapeutic delivery (94); thereby suggesting that PSC activation is an immunoediting characteristic of the TME in this malignancy. Importantly, as reviewed by Yao et al., the mesenchymal-epithelial transition (MET) could represent a relevant antibody-based therapeutic target (95). Rybrevant is an FDA-approved bispecific antibody targeting EGFR and MET receptor. Although it is indicated for treating locally advanced or metastatic non-small cell lung cancer with certain mutations, its therapeutic effect on different solid tumors, including PDAC, is currently being investigated in clinical trials (96)

Another important aspect of the tumor microenvironment in PaCa is its abundant extracellular matrix. The hyaluronic acid contained within is negatively charged, which allows its binding to large amounts of water. The latter results in high hydrostatic pressure and intersticial fluid pressure (97). As a result, therapy delivery is particularly problematic (98); furthermore, such TME behavior can also lead to hypoxia, a pervasive feature of cancer (99).

In terms of immune response in the PDAC context, a large amount of data indicates that PaCa TME is immunosuppressed at various levels (100). In keeping with this, Ene-Obong et al. showed for the first time that activated PSCs seemed to reduce migration of CD8^+^ T-cells to tumor-proximal stromal compartments (101). More recently, it has been suggested that T-cell suppression in PDAC may be promoted by several mechanisms: i) Treg accumulation, ii) M2 tumor-associated macrophages (TAMs), iii) myeloid-derived suppressor cell (MDSCs) and iv) fibroblast activation protein (FAP)^+^ fibroblasts, a type of stromal cell (93). Remarkably, Feig et al. reported latent immune responses and intratumoral accumulation of T-cells when FAP^+^ carcinoma-associated fibroblasts (CAFs) were removed and/or CXCR4 inhibitors were added to an *in vivo* model of human PDAC, suggesting that endogenous cytotoxic T-cells are not dysfunctional in this malignancy (19, 102).

Based on data from genetically engineered mouse models, it has been long thought that the dense desmoplastic nature of the TME in PDAC impaired T-cell infiltration. This led to consider PDAC tumors as ‘cold’ in terms of their immunogenicity. Recent studies on human PDAC tumors challenged this conviction by showing that T-cells are the dominant cell type found in the stroma of primary samples (103, 104). Carstens et al. demonstrated the presence of heterogenous populations of T-cells in PDAC with specific spatial distributions. They also showed that fibroblasts and type I collagen are not absolute inhibitors of T-cell infiltration (103).

The function of infiltrated T-cells is attenuated however by the coinfiltration of Treg cells, myeloid-derived suppressor cells and M2 macrophages; amongst other immunosuppressive mechanisms triggered by tumor cells and the TME. Therefore, the use of immunomodulating agents capable to address multiple and non-overlapping immune vulnerabilities is sorely needed. It is anticipated that the combination of checkpoint inhibitors -such as antibodies against PD1, PDL1, CTLA4, etc.- and therapeutic drugs able to prompt immune activation -such as chemotherapy, anti-CD40 antibodies, vaccines, adoptive T-cell therapy and BiTEs- will lead to better outcomes for PDAC patients (105–107).

## 4 Novel strategies for BiTE design

A recent study reported the use of oncolytic reovirus as a strategy to sensitize non-inflamed solid tumors in combination with CD3 bispecific antibodies (108). The researchers observed that reovirus and CD3-bsAbs led to the regression of large melanoma, pancreatic and breast cancer tumors *in vitro* by promoting local interferon response and strong T-cell influx. Notably, this combination therapy also induced regressions of distant tumors that were not injected with the virus. More recently, different combinations of oncolytic viruses (OVs) and BiTEs have been tested, including the injection of OVs harnessing BiTE encoding vectors (109). Notably, these BiTE encoding OVs have been associated with enhanced antigen presentation, T-cell proliferation, activation, and specific cytotoxicity against cancer cells. Furthermore, this combined approach addresses tumor heterogeneity, drug delivery, and T-cell infiltration, offering a comprehensive and effective solution. For a more detailed review on this subject, please refer to (109).

With regards to BiTE technology, efforts to overcome the limitation of their short serum half-life are being directed to conjugating the water-soluble polymer, poly(ethylene glycol) (PEG) to the antibody fragment (110). Alternatively, Amgen is developing half-life extended (HLE) BiTE molecules, which contain Fragment crystallizable (Fc) domains (111). The addition of Fc portions allows weekly administration of BiTEs instead of continuous infusion using pumps, which would make this therapeutic approach more convenient for the patients and their families.

The short half-life of BiTEs allows for a favorable safety profile when compared to other T-cell immunotherapies, such as CAR T-cells. Nevertheless, the potent effect of BiTEs could result in grade 3+ treatment related adverse events (TRAEs). While often manageable, there is a pressing need for safer T-cell engagers with improved tolerability and consistent efficacy. Recently, Granger et al., reported the addition of Autoregulation (AR) peptides -sensitive to granzyme B- to the linker sequence of NVG-222, a ROR1xCD3 HLE BiTE. Granzyme B is normally liberated by T-cells in order to eliminate their target. In this innovative, threshold-based approach, if granzyme B is encountered in high concentrations, AR peptides undergo proteolysis, resulting in destruction of the linker and, therefore, of the BiTE. NVG-222 has been pre-clinically tested, both *in vitro* and *in vivo*, resulting in no symptoms of toxicity observed in mice and the same level of tumor growth inhibition as their BiTE counterpart with no AR peptides (112). The authors intend to start clinical development of this construct for hematological and solid malignancies later this year.

As mentioned above, an important challenge in BiTE therapy against PDAC is the density and types of T-cells in the tumor, as their phenotype is difficult to predict. T-cell exhaustion and an immunosuppressive TME warrant further investigation on combinational approaches to boost the efficacy of BiTEs against pancreatic cancer (113).

## 5 Lessons from BiTEs on other types of cancer

In the next few paragraphs, a brief overview of BiTE antibodies in both hematologic and solid cancers is provided. For a more in-depth review, please refer to (114).

*Blinatumomab*: Also called MT103 and commercially known as Blincyto (Amgen); this binder is a CD19xCD3 non-IgG-like bispecific antibody. It targets both healthy and malignant B cells (CD19 antigen) and it was the first clinically tested and FDA-approved BiTE in 2014 for the treatment of CD19^+^ Philadelphia chromosome-negative (Ph^-^) relapsed and refractory B-ALL (36).

Preclinical studies showed evidence of cytotoxic activity against CD19^+^ B cells both *in vitro* and *in vivo*. Clinical trials in patients with relapsed or refractory B-cell precursor ALL showed significantly improved median overall survival (OS), which led to FDA approval for this indication as well. As reported by Martinelli et al., Blinatumomab was administered as a continuous intravenous (iv.) infusion of 9µg/day for the first 7 days followed by 28µg/day over a period of 4 weeks every 6 weeks for up to 5 cycles in the adult population (115). Some of the most common adverse events were pyrexia, headache, febrile neutropenia, and peripheral edema (116).

*Solitomab*: Also known as MT110, it is an EpCAMxCD3 BiTE antibody also developed by Amgen. EpCAM is a known biomarker for poor prognosis across a range of carcinomas and carcinosarcomas (117). It is highly expressed on the cell surface of various human carcinomas but at low levels on healthy epithelial tissue. In addition, EpCAM is involved in multiple cell functions such as cell migration, differentiation, signaling and proliferation, which makes EpCAM an attractive target for immunotherapy.

In preclinical studies, Solitomab demonstrated impressive antitumor activity against carcinosarcoma cell lines and primary tumor cells from patients with gynecologic carcinosarcomas (118). Lately, Kebenko et al. reported clinical data obtained from a multicenter Phase I study of Solitomab in patients with refractory solid tumors (colorectal, ovarian, gastric, non-small cell lung, small cell lung and hormone-refractory prostate cancers). BiTE therapy was administered by continuous intravenous infusion over at least 4 weeks. Unfortunately, treatment with Solitomab was associated with dose-limiting toxicities, including severe diarrhea and increased liver enzymes in 95% of patients treated with this BiTE. The latter treatment-related adverse events (grade ≥3) impeded dose escalation to potentially therapeutic levels (84).

Although BiTE technology has shown promising results in hematologic cancers, further investigation is needed in order to reach similar clinical benefit in solid malignancies. To date, two new BiTE conventional constructs have been tested on solid tumors in Phase I open label clinical trials: i) AMG 211, a carcinoembryonic antigen (CEA) in patients with relapse/refractory gastrointestinal adenocarcinoma (NCT02291614), and ii) Pasotuxizumab, a prostate-specific membrane antigen (PSMA) in patients with castration-resistant prostate cancer (NCT01723475). Additionally, other new conventional and HLE BiTE constructs are being evaluated in the clinic. For a list of clinical trials investigating BiTEs in pancreatic and other types of cancer, please see **Table 3**.

**Table 3:** Clinical trials investigating the therapeutic effect of BiTEs in haematologic and solid malignancies.

Name	Target	Disease	Trial	Developer	NCT
Blinatumomab (AMG 103, MT103)	CD19xCD3	ALL	Approved*	Amgen	NCT01466179
Solitomab (AMG 110, MT110)	EpCAMxCD3	Lung, gastric, colorectal, breast, prostate and ovarian cancer	Phase I (completed)	Amgen	NCT00635596
NVG-111	ROR1xCD3	Hematological malignancies	Phase I (recruiting)	NovalGen	NCT04763083
AMG 160	PSMAxCD3 (HLE)	mCRPC, Prostate cancer	Phase I (terminated)	Amgen	NCT03792841, NCT04631601
AMG 199	MUC17xCD3 (HLE)	Gastric and gastroesophageal junction	Phase I (terminated)	Amgen	NCT04117958
AMG 211, MEDI-565	CEAxCD3	Gastrointestinal adenocarcinomas	Phase I (completed)	Amgen	NCT02291614
Pasotuxizumab (AMG 212, MT112)	PSMAxCD3	Prostate cancer	Phase I (completed)	Bayer	NCT01723475
AMG 305	CDH3xCD3/ MSLNxCD3 (HLE)**	Solid tumors	Phase I (recruiting)	Amgen	NCT05800964
AMG 330	CD33xCD3	AML	Phase I (terminated)	Amgen	NCT02520427
AMG 420	BCMAxCD3	Relapsed/refractory MM	Phase I (completed with results)	Amgen	NCT03836053
AMG 427	FLT3xCD3 (HLE)	Relapsed/refractory AML	Phase I (terminated)	Amgen	NCT03541369
AMG 562	CD19xCD3 (HLE)	DLBCL, MCL, FL	Phase I (terminated with results)	Amgen	NCT03571828
AMG 596	EGFRvIIIxCD3	Glioblastoma	Phase I (completed)	Amgen	NCT03296696
AMG 673	CD33xCD3 (HLE)	Relapsed/refractory AML	Phase I (terminated with results)	Amgen	NCT03224819
AMG 701	BCMAxCD3 (HLE)	Relapsed/refractory MM	Phase I (terminated/available)	Amgen	NCT03287908, NCT05256446
Tarlatamab (AMG 757)	DLL3xCD3 (HLE)	Small-cell lung cancer, neuroendocrine prostate cancer, Limited stage small-cell lung cancer	Phase I (recruiting/active, not recruiting) Phase III (recruiting)	Amgen	NCT03319940, NCT04702737, NCT06117774
AMG 794	CDLN6xCD3 (HLE)	Solid tumors	Phase I (active, not recruiting)	Amgen	NCT05317078
AMG 910	CLDN18.2xCD3 (HLE)	Gastric cancer	Phase I (terminated with results)	Amgen	NCT04260191

AMG, Amgen identification number; AML, Acute myeloid leukaemia; BCMA, B-cell maturation antigen; CDH3, P-cadherin; CDLN6, Claudin-6 ; CLDN18.2, Claudin-18 isoform 2; DLBCL, Diffuse large B-cell lymphoma; DLL3, δ-like protein 3; EGFRvIII, epidermal growth factor receptor VIII; FL, follicular lymphoma; FLT3, FMS-like tyrosine kinase 3; HLE, Half-life extended BiTE format; MCL, Mantle cell lymphoma; mCRPC, Metastatic castrate-resistant prostate cancer; MM, Multiple myeloma; MSLN, Mesothelin; MUC17, Mucin 17; NCT, National Clinical Trials identification number (clinicaltrials.gov); PSMA, Prostate-specific membrane antigen.

*Apart from ALL, Blinatumomab is being tested in various hematological cancers as a single agent and in combination with other drugs. For further details, please visit: https://clinicaltrials.gov/ct2/results?cond=&term=blinatumomab&cntry=&state=&city=&dist.

**Dual targeting BiTE.

In the case of AMG 211, 44 patients with either colorectal carcinoma, pancreatic cancer, cholangiocarcinoma, esophageal or appendix adenocarcinoma were treated with continuous iv. administration for 7, 14 or 28 days in repeated cycles until either of the following events were reported: i) confirmed disease progression, ii) occurrence of a dose-limiting toxicity (DLT) or iii) discontinuation for other reasons. Treatment with AMG 211 was stopped due to disease progression in 73% of participants (33 patients), adverse events in 16% (7 patients), patient’s request and other reasons in 4% (2 patients). In general, although initial changes in inflammatory and tumor markers were detected accompanied by an acceptable safety profile, the study was discontinued after observation of anti-AMG 211 antibodies in all patients treated at high doses of >3.2mg (119).

With regards to Pasotuxizumab, results of a first-in-human, multicenter, dose-escalation study in patients with metastatic castration-resistant prostate cancer (mCRPC) refractory to standard therapy showed that patients treated with subcutaneous Pasotuxizumab once a day developed antidrug antibodies. Continuous iv. infusion was then assessed, although the maximum tolerated dose (MTD) could not be determined due to early termination. The subcutaneous MTD was 172μg/d. Prostate-Specific Antigen (PSA) responders were observed (>50% PSA decline n=12), including two long-term responders. Overall, Pasotuxizumab treatment showed clinical safety in advanced castration-resistant prostate cancer patients. Importantly, data provides evidence of BiTE monotherapy efficacy in solid tumors (120).

As mentioned above, a recent innovation to the BiTE format has been introduced with the HLE design, which consists of fusing an Fc portion to a conventional BiTE molecule. Due to their increased half-life, HLE-BiTEs specific for different targets in both hematological and solid cancers are now being tested in clinical trials. Unfortunately, most of these clinical studies had to be terminated due to safety reasons or because of a business decision. Nonetheless, Tarlatamab (AMG 757), a HLE BiTE targeting the protein delta-like ligand 3 (DLL3) and CD3, has recently been granted Breakthrough Therapy Designation by the FDA (111) and FDA priority review (121, 122). DLL3 is a part of the Delta/Serrate/Lag2 (DSL) Notch receptor ligand family and plays a key role in Notch signaling, which influences various cellular processes including differentiation, proliferation, survival, and apoptosis. Moreover, it has been found that DLL3 is associated with various solid malignancies, including lung, liver, and pancreatic cancer (123). Tarlatamab, a DLL3xCD3 HLE BiTE, is currently in Phase III clinical trials for treating small-cell lung cancer (NCT06117774). On May 2024, Tarlatamab was granted FDA-accelerated approval for extensive stage small cell lung cancer, becoming the first and so far, the only BiTE therapy for a major solid tumor (124).

Another BiTE to watch is NVG-222, a next-generation HLE ROR1xCD3 BiTE (112). Not yet in the clinic, this BiTE builds on the technology used on NovalGen’s first T-cell engager, NVG-111, a ROR1xCD3 BiTE, currently in Phase I clinical trials for hematological malignancies. The novelty of NVG-222 lies in its half-life extended format and its capability for self-destruction in the presence of high levels of granzyme B. Granger et al. reported no toxicity symptoms on tumor-bearing mice treated with this drug in pre-clinical studies. Although the authors did not disclose what kind of cancer was tested, ROR1 overexpression is found on both hematological and solid cancers, including PaCa (19), a tantalizing prospect.

## 6 Discussion

Better understanding of the immune system and its interactions with cancer cells and the TME has resulted in the advancement of the immunotherapy field. Certainly, immunotherapy has rapidly emerged as a promising approach for the treatment of cancer patients since remarkable clinical benefit has been observed with checkpoint inhibitors (CTLA-4, PD1 and PDL1 antibodies), adoptive cell therapy (TILs, CAR T-cells), and immunomodulating agents (chemotherapy, small molecules, etc.).

Antibody derivatives such as BiTEs are a major example of T-cell based therapies developed upon increasing knowledge of T-cell activation in the cancer context. These non-IgG-like bispecific molecules are composed of only two scFvs joined by a flexible linker, whereby one binds to CD3 (T-cells) and the other one to TAA (cancer cells).

A key advantage of BiTEs is their ability to redirect T-cells to tumor sites. Moreover, simultaneous binding of BiTEs to T-cells and cancer cells is required to elicit T-cell activation and consequent tumor cell lysis, preventing therefore undesired anergy. Consistent with this, preclinical data both *in vitro* and *in vivo* have shown the remarkable cytotoxic potency of BiTEs at subnanomolar concentrations. Similarly, Blinatumomab, the first FDA-approved BiTE, has attained impressive clinical responses in patients with ALL and other B-cell malignancies in Phase I/II clinical trials.

Additionally, BiTEs provide the added benefit of stepwise dose escalation and rapid cessation of treatment should toxicity occur. Some of the most common adverse events observed upon BiTE therapy are: fever, nausea, vomiting, lymphopenia, leukopenia, hepatotoxicity, CRS, amongst others. Nevertheless, these symptoms are reversible once treatment is ended.

For solid cancers, other BiTEs have been developed, including those targeting EpCAM (for carcinomas and carcinosarcomas), PSMA (castrate-resistant prostate cancer) and CEA (gastrointestinal adenocarcinomas). Although the EpCAM BiTE was tested on pancreatic cancer cells and CSCs at a preclinical level with encouraging results, Phase I clinical studies did not include PaCa patients. In contrast, CEA BiTEs were investigated at a preclinical level followed by a Phase I clinical trial that included patients with PaCa; unfortunately, no objective responses were observed.

In an effort to increase the stability and solubility of BiTEs in patients, a new design of Half-Life Extended (HLE) BiTEs have emerged, whereby BiTE molecules are coupled to an Fc portion. HLE-BiTEs targeting several TAA have been recently under study. Unfortunately, most of them have not progressed beyond Phase I clinical trials. It is hypothesized that the increased half-life of the molecule might have an undesired effect on adverse events. Nevertheless, Tarlatamab (AMG 757), a DLL3xCD3 HLE-BiTE, is being tested in patients with small cell lung cancer in Phase III clinical trials. On December 2023 was granted Priority Review by the FDA (122) and earlier this year, it became the first BiTE for solid cancers, indicated for extensive small cell lung cancer (124). Importantly, DLL3 has been found to be overexpressed on PaCa cells compared to human pancreatic epithelial cells. Although the location of DLL3 expression on the pancreatic cancer cell is still unclear, its absence results in growth inhibition (123) This finding warrants further investigation on this molecule as a druggable target in PaCa.

In terms of safety, NVG-222 might represent a balanced combination of increased half-life and enhanced safety profile due to the presence of autoregulating (AR), threshold-based peptides sensitive to high levels of granzyme B (112). Notably, this AR technology could be applied to different types of BiTEs, leading to improved safety and efficacy outcomes of other BiTE constructs. Importantly, NVG-222 is an anti-ROR1 T-cell engager, which could prove useful in a variety of hematological and solid tumors, including PaCa.

Pancreatic cancer is an aggressive and chemoresistant malignancy with a very poor prognosis (65). Genetic instability, local immunosuppressive microenvironment, desmoplastic stromal changes and the presence of highly persistent CSCs are some of the main hurdles that make PaCa a challenging disease to treat. Even with current therapies, a median survival of less than 6 months and overall 5-year survival rates of 1-5% are obtained (70).

Recent advances in immunotherapy have opened new possibilities for the treatment of PaCa. As reviewed by Chen et al., specific immunotherapies such as vaccines with autologous tumor cells, TAA-specific MAbs, and antibodies able to mediate an immune response including BiTEs significantly improved OS and augmented the immune response of PaCa patients (125). Currently, there are two FDA-approved immunotherapy options for a small subset of patients with pancreatic cancer: Dostarlimab (Jemperli) and Pembrolizumab (Keytruda). Both antibodies are immunomodulators targeting the PD-1/PD-L1 pathway; approved for subsets of patients with advanced pancreatic cancer that has DNA mismatch repair deficiency (dMMR), and those with high microsatellite instability (MSI-H), DNA mismatch repair deficiency (dMMR), or high tumor mutational burden (TMB-H) (67). It is envisaged that drug combinations able to evade the immunosuppressive TME, promote T-cell activation and target tumorigenic CSCs will prove instrumental to PDAC therapy. Certainly, the recent approval of Tarlatamab, the first BiTE for solid tumors targeting DLL3, a protein found in small cell lung cancer and in pancreatic cancer, opens the possibility of future studies of this BiTE in PaCa.

In conclusion, BiTEs represent a major advancement in cancer immunotherapy. Nevertheless, further improvement in the management of toxicities coupled with better targets expressed on CSCs and malignant cells but not healthy tissue will enhance the great promise that BiTE technology offers for patients. Combinations of different targeted and immunotherapeutic strategies with currently available drugs warrant further investigation in the clinic, particularly in those patients suffering from cancers where survival rates are dismal, such as pancreatic cancer.

## References

[1] Hanahan D Weinberg RA. Hallmarks of cancer: The next generation. Cell. (2011) 144. doi: 10.1016/j.cell.2011.02.013 21376230

[2] Hanahan D. Hallmarks of cancer: new dimensions. Cancer Discov. (2022) 12. doi: 10.1158/2159-8290.CD-21-1059 35022204

[3] Coley WBMD. The treatment of Malignant tumors by repeated inoculations of erysipelas: With a report of ten original cases. Clin Orthop Relat Res. (1893) 262). 1984929

[4] Ehrlich P. Über Den Jetzigen Stand Der Karzinomforschung Beiträge zur experimentellen. Pathologie und Chemotherapie. (1909). doi: 10.1002/cber.19090420105

[5] Lawrence HS. Cellular and Humoral Aspects of the Hypersensitive States: A Symposium Held at the New York Academy of Medicine. Hoeber PB, editor. New York, USA: New York, P.B. Hoeber (1959).

[6] Burnet FM. Immunological aspects of Malignant disease. Lancet. (1967) 289. doi: 10.1016/S0140-6736(67)92837-1 4165129

[7] Shankaran V Ikeda H Bruce AT White JM Swanson PE Old LJ. IFNgamma and lympohcytes prevent primary tomour development and shape tomour immunogenicity. Nature. (2001) 410. doi: 10.1038/35074122 11323675

[8] Dunn GP Old LJ Schreiber RD. The three Es of cancer immunoediting. Annu Rev Immunol. (2004) 22. doi: 10.1146/annurev.immunol.22.012703.104803 15032581

[9] O’Donnell JS Teng MWL Smyth MJ. Cancer immunoediting and resistance to T cell-based immunotherapy. Nat Rev Clin Oncol. (2019) 16. doi: 10.1038/s41571-018-0142-8 30523282

[10] Pardoll DM. The blockade of immune checkpoints in cancer immunotherapy. Nat Rev Cancer. (2012) 12. doi: 10.1038/nrc3239 PMC4856023 22437870

[11] Schwartz RH. Costimulation of T lymphocytes: the role of CD28, CTLA-4, and B7/BB1 in interleukin-2 production and immunotherapy. Cell. (1992) 71. doi: 10.1016/S0092-8674(05)80055-8 1335362

[12] Chen L Flies DB. Molecular mechanisms of T cell co-stimulation and co-inhibition. Nat Rev Immunol. (2013) 13. doi: 10.1038/nri3405 PMC3786574 23470321

[13] Le Mercier I Lines JL Noelle RJ. Beyond CTLA-4 and PD-1, the generation Z of negative checkpoint regulators. Front Immunol. (2015) 6. doi: 10.3389/fimmu.2015.00418 PMC4544156 26347741

[14] Chen S Li J Li Q Wang Z. Bispecific antibodies in cancer immunotherapy. Hum Vaccin Immunother. (2016) 12:2491–500. doi: 10.1080/21645515.2016.1187802 PMC5084997 27249163

[15] Dahlén E Veitonmäki N Norlén P. Bispecific antibodies in cancer immunotherapy. Ther Adv Vaccines Immunotherapy. (2018) 6. doi: 10.1177/2515135518763280 PMC5933537 29998217

[16] Khalil DN Smith EL Brentjens RJ Wolchok JD. The future of cancer treatment: Immunomodulation, CARs and combination immunotherapy. Nat Rev Clin Oncol. (2016) 13. doi: 10.1038/nrclinonc.2016.25 PMC5558237 27118494

[17] Chen DS Mellman I. Oncology meets immunology: The cancer-immunity cycle. Immunity. (2013) 39. doi: 10.1016/j.immuni.2013.07.012 23890059

[18] Voena C Chiarle R. Advances in cancer immunology and cancer immunotherapy. Discovery Med. (2016) 21:125–33. 27011048

[19] Paredes-Moscosso SR. ROR1 as a target for cancer immunotherapy. LONDON: University College London (2017).

[20] Lowdell MW Thomas A. The expanding role of the clinical haematologist in the new world of advanced therapy medicinal products. Br J Haematology. (2017) 176. doi: 10.1111/bjh.2017.176.issue-1 27748517

[21] Couzin-Frankel J. Breakthrough of the year 2013. Cancer immunotherapy. Science. (2013) 342. doi: 10.1126/science.342.6165.1432 24357284

[22] Köhler G Milstein C. Continuous cultures of fused cells secreting antibody of predefined specificity. Nature. (1975) 256. doi: 10.1038/256495a0 1172191

[23] Strebhardt K Ullrich A. Paul Ehrlich’s magic bullet concept: 100 Years of progress. Nat Rev Cancer. (2008) 8. doi: 10.1038/nrc2394 18469827

[24] Ohlin M Zouali M. The human antibody repertoire to infectious agents: Implications for disease pathogenesis. Mol Immunol. (2003) 40. doi: 10.1016/S0161-5890(03)00099-3 12909126

[25] Nelson PN. Demystified…: monoclonal antibodies. Mol Pathology. (2000) 53:111–7. doi: 10.1136/mp.53.3.111 PMC1186915 10897328

[26] Scott AM Wolchok JD Old LJ. Antibody therapy of cancer. Nat Rev Cancer. (2012) 12. doi: 10.1038/nrc3236 22437872

[27] Loisel S André PA Golay J Buchegger F Kadouche J Cérutti M. Antitumour effects of single or combined monoclonal antibodies directed against membrane antigens expressed by human B cells leukaemia. Mol Cancer. (2011) 10. doi: 10.1186/1476-4598-10-42 PMC3103468 21504579

[28] Sathyanarayanan V Neelapu SS. Cancer immunotherapy: Strategies for personalization and combinatorial approaches. Mol Oncol. (2015) 9. doi: 10.1016/j.molonc.2015.10.009 PMC4684975 26548534

[29] Lum LG Thakur A Choi M Deol A Kondadasula V Schalk D. Clinical and immune responses to anti-CD3 x anti-EGFR bispecific antibody armed activated T cells (EGFR BATs) in pancreatic cancer patients. Oncoimmunology. (2020) 9. doi: 10.1080/2162402X.2020.1773201 PMC7480816 32939319

[30] Nicholas NS Apollonio B Ramsay AG. Tumor microenvironment (TME)-driven immune suppression in B cell Malignancy. Biochim Biophys Acta - Mol Cell Res. (2016) 1863. doi: 10.1016/j.bbamcr.2015.11.003 26554850

[31] Lee CS Cragg M Glennie M Johnson P. Novel antibodies targeting immune regulatory checkpoints for cancer therapy. Br J Clin Pharmacol. (2013) 76. doi: 10.1111/bcp.2013.76.issue-2 PMC3731598 23701301

[32] Berman D Korman A Peck R Feltquate D Lonberg N Canetta R. The development of immunomodulatory monoclonal antibodies as a new therapeutic modality for cancer: The Bristol-Myers Squibb experience. Pharmacol Ther. (2015) 148. doi: 10.1016/j.pharmthera.2014.11.017 25476108

[33] Beck A Wurch T Bailly C Corvaia N. Strategies and challenges for the next generation of therapeutic antibodies. Nat Rev Immunol. (2010) 10. doi: 10.1038/nri2747 20414207

[34] Bispecific Antibodies: An Area of Research and Clinical Applications (2024). Available online at: https://www.fda.gov/drugs/spotlight-cder-science/bispecific-antibodies-area-research-and-clinical-applications (accessed May 03, 2024).

[35] Reichert J. Bispecific antibodies come to the fore (2020). Available online at: https://www.linkedin.com/pulse/bispecific-antibodies-come-fore-janice-reichert/ (accessed May 07, 2024).

[36] Krishnamurthy A Jimeno A. Bispecific antibodies for cancer therapy: A review. Pharmacol Ther. (2018) 185. doi: 10.1016/j.pharmthera.2017.12.002 29269044

[37] Sedykh SE Prinz VV Buneva VN Nevinsky GA. Bispecific antibodies: Design, therapy, perspectives. Drug Design Dev Ther. (2018) 12. doi: 10.2147/DDDT.S151282 PMC5784585 29403265

[38] Suryadevara CM Gedeon PC Sanchez-Perez L Verla T Alvarez-Breckenridge C Choi BD. Are BiTEs the “missing link” in cancer therapy? OncoImmunology. (2015) 4. doi: 10.1080/2162402X.2015.1008339 PMC4485829 26155413

[39] Baeuerle PA Kufer P Bargou R. BiTE: Teaching antibodies to engage T-cells for cancer therapy. Curr Opin Mol Ther. (2009) 11. 19169956

[40] Stieglmaier J Benjamin J Nagorsen D. Utilizing the BiTE (bispecific T-cell engager) platform for immunotherapy of cancer. Expert Opin Biol Ther. (2015) 15. doi: 10.1517/14712598.2015.1041373 25971805

[41] Kipriyanov SM Moldenhauer G Schuhmacher J Cochlovius B Von Der Lieth CW Matys ER. Bispecific tandem diabody for tumor therapy with improved antigen binding and pharmacokinetics. J Mol Biol. (1999) 293. doi: 10.1006/jmbi.1999.3156 10512714

[42] Bohlen H Manzke O Patel B Moldenhauer G Dörken B von Fliedner V. Cytolysis of leukemic B-cells by T-cells activated via two bispecific antibodies. Cancer Res. (1993) 53. 7689932

[43] Huehls AM Coupet TA Sentman CL. Bispecific T-cell engagers for cancer immunotherapy. Immunol Cell Biol. (2015) 93. doi: 10.1038/icb.2014.93 PMC4445461 25367186

[44] Dreier T Lorenczewski G Brandl C Hoffmann P Syring U Hanakam F. Extremely potent, rapid and costimulation-independent cytotoxic T-cell response against lymphoma cells catalyzed by a single-chain bispecific antibody. Int J Cancer. (2002) 100. doi: 10.1002/ijc.v100:6 12209608

[45] Gonnermann D Oberg HH Lettau M Peipp M Bauerschlag D Sebens S. Galectin-3 released by pancreatic ductal adenocarcinoma suppresses γδ T cell proliferation but not their cytotoxicity. Front Immunol. (2020) 11. doi: 10.3389/fimmu.2020.01328 PMC7338555 32695112

[46] Brischwein K Parr L Pflanz S Volkland J Lumsden J Klinger M. Strictly target cell-dependent activation of T cells by bispecific single-chain antibody constructs of the BiTE class. J Immunotherapy. (2007) 30. doi: 10.1097/CJI.0b013e318156750c 18049331

[47] Amann M DÁrgouges S Lorenczewski G Brischwein K Kischel R Lutterbuese R. Antitumor activity of an EpCAM/CD3-bispecific BiTE antibody during long-term treatment of mice in the absence of t-cell anergy and sustained cytokine release. J Immunotherapy. (2009) 32. doi: 10.1097/CJI.0b013e3181a1c097 19609237

[48] Nagorsen D Baeuerle PA. Immunomodulatory therapy of cancer with T cell-engaging BiTE antibody blinatumomab. Exp Cell Res. (2011) 317. doi: 10.1016/j.yexcr.2011.03.010 21419116

[49] Hoffmann P Hofmeister R Brischwein K Brandl C Crommer S Bargou R. Serial killing of tumor cells by cytotoxic T cells redirected with a CD19-/CD3-bispecific single-chain antibody construct. Int J Cancer. (2005) 115. doi: 10.1002/ijc.v115:1 15688411

[50] Newman MJ Benani DJ. A review of blinatumomab, a novel immunotherapy. J Oncol Pharm Pract. (2016) 22. doi: 10.1177/1078155215618770 26607163

[51] Choi BD Gedeon PC Sanchez-Perez L Bigner DD Sampson JH. Regulatory T cells are redirected to kill glioblastoma by an EGFRviii-targeted bispecific antibody. Oncoimmunology. (2013) 2. doi: 10.4161/onci.26757 PMC3891636 24475376

[52] Brischwein K Schlereth B Guller B Steiger C Wolf A Lutterbuese R. MT110: A novel bispecific single-chain antibody construct with high efficacy in eradicating established tumors. Mol Immunol. (2006) 43. doi: 10.1016/j.molimm.2005.07.034 16139892

[53] Haas C Krinner E Brischwein K Hoffmann P Lutterbüse R Schlereth B. Mode of cytotoxic action of T cell-engaging BiTE antibody MT110. Immunobiology. (2009) 214:441–53. doi: 10.1016/j.imbio.2008.11.014 19157637

[54] Dhimolea E Reichert JM. World bispecific antibody summit, september 27-28, 2011, Boston, MA. MAbs. (2012). doi: 10.4161/mabs.4.1.18821 PMC3338936 22327426

[55] Portell CA Wenzell CM Advani AS. Clinical and pharmacologic aspects of blinatumomab in the treatment of B-cell acute lymphoblastic leukemia. Clin Pharmacology: Adv Appl. (2013) 5. doi: 10.2147/CPAA.S42689 PMC3650887 23671399

[56] Topp MS Gökbuget N Zugmaier G Degenhard E Goebeler ME Klinger M. Long-term follow-up of hematologic relapse-free survival in a phase 2 study of blinatumomab in patients with MRD in B-lineage ALL. Blood. (2012) 120. doi: 10.1182/blood-2012-07-441030 23024237

[57] Köhnke T Krupka C Tischer J Knösel T Subklewe M. Increase of PD-L1 expressing B-precursor ALL cells in a patient resistant to the CD19/CD3-bispecific T cell engager antibody blinatumomab. J Hematol Oncol. (2015) 8. doi: 10.1186/s13045-015-0213-6 PMC4599591 26449653

[58] Duell J Dittrich M Bedke T Mueller T Eisele F Rosenwald A. Frequency of regulatory T cells determines the outcome of the T-cell-engaging antibody blinatumomab in patients with B-precursor ALL. Leukemia. (2017) 31. doi: 10.1038/leu.2017.41 PMC5629361 28119525

[59] Klinger M Brandl C Zugmaier G Hijazi Y Bargou RC Topp MS. Immunopharmacologic response of patients with B-lineage acute lymphoblastic leukemia to continuous infusion of T cell-engaging CD19/CD3-bispecific BiTE antibody blinatumomab. Blood. (2012) 119. doi: 10.1182/blood-2012-01-400515 22592608

[60] Topp MS Kufer P Gökbuget N Goebeler M Klinger M Neumann S. Targeted therapy with the T-cell - Engaging antibody blinatumomab of chemotherapy-refractory minimal residual disease in B-lineage acute lymphoblastic leukemia patients results in high response rate and prolonged leukemia-free survival. J Clin Oncol. (2011) 29. doi: 10.1200/JCO.2010.32.7270 21576633

[61] Nisonoff A Rivers MM. Recombination of a mixture of univalent antibody fragments of different specificity. Arch Biochem Biophysics. (1961) 93. doi: 10.1016/0003-9861(61)90296-X 13729244

[62] Zhang X Yang Y Fan D Xiong D. The development of bispecific antibodies and their applications in tumor immune escape. Exp Hematol Oncol. (2017) 6. doi: 10.1186/s40164-017-0072-7 PMC5414286 28469973

[63] Löffler A Kufer P Lutterbüse R Zettl F Daniel PT Schwenkenbecher JM. A recombinant bispecific single-chain antibody, CD19 x CD3, induces rapid and high lymphoma-directed cytotoxicity by unstimulated T lymphocytes. Blood. (2000) 95. 10706880

[64] Brinkmann U Kontermann RE. The making of bispecific antibodies. MAbs. (2017) 9. doi: 10.1080/19420862.2016.1268307 PMC5297537 28071970

[65] Siegel RL Miller KD Wagle NS Jemal A. Cancer statistics, 2023. CA Cancer J Clin. (2023) 73. doi: 10.3322/caac.21763 36633525

[66] Ilic I Ilic M. International patterns in incidence and mortality trends of pancreatic cancer in the last three decades: A joinpoint regression analysis. World J Gastroenterol. (2022) 28:4698–715. doi: 10.3748/wjg.v28.i32.4698 PMC9476884 36157927

[67] Cancer Research Institute. How is Immunotherapy for Pancreatic Cancer Changing the Outlook for Patients . Available online at: https://www.cancerresearch.org/cancer-types/pancreatic-cancer (accessed April 30, 2024).

[68] IARC. Cancer Tomorrow - Globocan 2022 (2024). Available online at: https://gco.iarc.who.int (accessed May 03, 2024).

[69] Muniraj T Jamidar PA Aslanian HR. Pancreatic cancer: A comprehensive review and update. Disease-a-Month. (2013) 59. doi: 10.1016/j.disamonth.2012.10.002 24183261

[70] Ercan G Karlitepe A Ozpolat B. Pancreatic cancer stem cells and therapeutic approaches. Anticancer Res. (2017) 37. doi: 10.21873/anticanres.11628 28551612

[71] Xu JW Wang L Cheng YG Zhang GY Hu SY Zhou B. Immunotherapy for pancreatic cancer: A long and hopeful journey. Cancer Lett. (2018) 425. doi: 10.1016/j.canlet.2018.03.040 29605510

[72] Costello E Greenhalf W Neoptolemos JP. New biomarkers and targets in pancreatic cancer and their application to treatment. Nat Rev Gastroenterol Hepatol. (2012) 9. doi: 10.1038/nrgastro.2012.119 22733351

[73] Ciliberto D Botta C Correale P Rossi M Caraglia M Tassone P. Role of gemcitabine-based combination therapy in the management of advanced pancreatic cancer: A meta-analysis of randomised trials. Eur J Cancer. (2013) 49. doi: 10.1016/j.ejca.2012.08.019 22989511

[74] Nakai Y Isayama H Sasaki T Sasahira N Tsujino T Toda N. A multicentre randomised phase II trial of gemcitabine alone vs gemcitabine and S-1 combination therapy in advanced pancreatic cancer: GEMSAP study. Br J Cancer. (2012) 106. doi: 10.1038/bjc.2012.183 PMC3388559 22555398

[75] Zhou G Chiu D Qin D Niu L Cai J He L. Expression of CD44v6 and integrin-β1 for the prognosis evaluation of pancreatic cancer patients after cryosurgery. Diagn Pathol. (2013) 8:146. doi: 10.1186/1746-1596-8-146 24004467 PMC3846138

[76] Warshaw AL Fernández-del Castillo C. Pancreatic carcinoma. New Engl J Med. (1992) 326:455–65. doi: 10.1056/NEJM199202133260706 1732772

[77] Wolfgang CL Herman JM Laheru DA Klein AP Erdek MA Fishman EK. Recent progress in pancreatic cancer. CA Cancer J Clin. (2013) 63:318–48. doi: 10.3322/caac.21190 PMC3769458 23856911

[78] Zhang Z Song J Xie C Pan J Lu W Liu M. Pancreatic cancer: recent progress of drugs in clinical trials. AAPS J. (2021) 23. doi: 10.1208/s12248-021-00556-2 33580411

[79] Qiu H Fang X Luo Q Ouyang G. Cancer stem cells: A potential target for cancer therapy. Cell Mol Life Sci. (2015) 72. doi: 10.1007/s00018-015-1920-4 PMC11113644 25967289

[80] Przepiorka D Ko CW Deisseroth A Yancey CL Candau-Chacon R Chiu HJ. FDA approval: blinatumomab. Clin Cancer Res. (2015) 21. doi: 10.1158/1078-0432.CCR-15-0612 26374073

[81] Baeuerle PA Gires O. EpCAM (CD326) finding its role in cancer. Br J Cancer. (2007). doi: 10.1038/sj.bjc.6603494 PMC2360029 17211480

[82] Munz M Baeuerle PA Gires O. The emerging role of EpCAM in cancer and stem cell signaling. Cancer Res. (2009) 69. doi: 10.1158/0008-5472.CAN-09-0654 19584271

[83] Cioffi M Dorado J Baeuerle PA Heeschen C. EpCAM/CD3-bispecific T-cell engaging antibody MT110 eliminates primary human pancreatic cancer stem cells. Clin Cancer Res. (2012) 18. doi: 10.1158/1078-0432.CCR-11-1270 22096026

[84] Kebenko M Goebeler ME Wolf M Hasenburg A Seggewiss-Bernhardt R Ritter B. A multicenter phase 1 study of solitomab (MT110, AMG 110), a bispecific EpCAM/CD3 T-cell engager (BiTE®) antibody construct, in patients with refractory solid tumors. Oncoimmunology. (2018) 7. doi: 10.1080/2162402X.2018.1450710 PMC6136859 30221040

[85] Pishvaian M Morse MA McDevitt J Norton JD Ren S Robbie GJ. Phase 1 dose escalation study of MEDI-565, a bispecific T-cell engager that targets human carcinoembryonic antigen, in patients with advanced gastrointestinal adenocarcinomas. Clin Colorectal Cancer. (2016) 15. doi: 10.1016/j.clcc.2016.07.009 27591895

[86] Zhang H Yang Z Zhu X Li J Gao Y Zhang Y. Phase I trial of hypoxia-responsive CEA CAR-T cell therapy in patients with heavily pretreated solid tumor via intraperitoneal or intravenous transfusion. J Clin Oncol. (2024) 42:3514–4. doi: 10.1200/JCO.2024.42.16_suppl.3514

[87] Gohil SH Paredes-Moscosso SR Harrasser M Vezzalini M Scarpa A Morris E. An ROR1 bi-specific T-cell engager provides effective targeting and cytotoxicity against a range of solid tumors. Oncoimmunology. (2017) 6. doi: 10.1080/2162402X.2017.1326437 PMC5543882 28811962

[88] Radziejewski C. Clinical-stage ROR1xCD3 bispecific antibodies with potential for broad cancer specificity (2022). Available online at: https://www.antibodysociety.org/bispecific-antibodies/ (accessed April 30, 2022).

[89] Townsend W Leong S Shah M Batten T Tucker D Pottinger B. Time limited exposure to a ROR1 targeting bispecific T cell engager (NVG-111) leads to durable responses in subjects with relapsed refractory chronic lymphocytic leukemia (CLL) and mantle cell lymphoma (MCL). Blood. (2023) 142. doi: 10.1182/blood-2023-188607

[90] Giraldo NA Sanchez-Salas R Peske JD Vano Y Becht E Petitprez F. The clinical role of the TME in solid cancer. Br J Cancer. (2019) 120. doi: 10.1038/s41416-018-0327-z PMC6325164 30413828

[91] Ceyhan GO Friess H. Pancreatic disease in 2014: Pancreatic fibrosis and standard diagnostics. Nat Rev Gastroenterol Hepatol. (2015) 12. doi: 10.1038/nrgastro.2014.234 25560846

[92] Dvorak HF. Tumors: wounds that do not heal. Similarities between tumor stroma generation and wound healing. N Engl J Med. (1986) 315. doi: 10.1056/NEJM198612253152606 3537791

[93] Makohon-Moore A Iacobuzio-Donahue CA. Pancreatic cancer biology and genetics from an evolutionary perspective. Nat Rev Cancer. (2016) 16. doi: 10.1038/nrc.2016.66 PMC5739515 27444064

[94] Sherman MH Yu RT Engle DD Ding N Atkins AR Tiriac H. Vitamin D receptor-mediated stromal reprogramming suppresses pancreatitis and enhances pancreatic cancer therapy. Cell. (2014) 159. doi: 10.1016/j.cell.2014.08.007 PMC4177038 25259922

[95] Yao HP Hudson R Wang MH. Progress and challenge in development of biotherapeutics targeting MET receptor for treatment of advanced cancer. Biochim Biophys Acta - Rev Cancer. (2020) 1874. doi: 10.1016/j.bbcan.2020.188425 32961258

[96] A Modular Multi-Basket Trial to Improve Personalized Medicine in Cancer Patients (Basket of Baskets) (BoB) . Available online at: https://clinicaltrials.gov/study/NCT03767075?term=amivantamab-vmjw&page=3&rank=23 (accessed January 18, 2024).

[97] Stromnes IM DelGiorno KE Greenberg PD Hingorani SR. Stromal reengineering to treat pancreas cancer. Carcinogenesis. (2014) 35:1451–60. doi: 10.1093/carcin/bgu115 PMC4076816 24908682

[98] Provenzano PP Cuevas C Chang AE Goel VK Von Hoff DD Hingorani SR. Enzymatic targeting of the stroma ablates physical barriers to treatment of pancreatic ductal adenocarcinoma. Cancer Cell. (2012) 21. doi: 10.1016/j.ccr.2012.01.007 PMC3371414 22439937

[99] Olive KP Jacobetz MA Davidson CJ Gopinathan A McIntyre D Honess D. Inhibition of Hedgehog signaling enhances delivery of chemotherapy in a mouse model of pancreatic cancer. Sci (1979). (2009) 324. doi: 10.1126/science.1171362 PMC2998180 19460966

[100] Clark CE Hingorani SR Mick R Combs C Tuveson DA Vonderheide RH. Dynamics of the immune reaction to pancreatic cancer from inception to invasion. Cancer Res. (2007) 67. doi: 10.1158/0008-5472.CAN-07-0175 17909062

[101] Ene-Obong A Clear AJ Watt J Wang J Fatah R Riches JC. Activated pancreatic stellate cells sequester CD8+ T cells to reduce their infiltration of the juxtatumoral compartment of pancreatic ductal adenocarcinoma. Gastroenterology. (2013) 145. doi: 10.1053/j.gastro.2013.07.025 PMC3896919 23891972

[102] Feig C Jones JO Kraman M Wells RJB Deonarine A Chan DS. Targeting CXCL12 from FAP-expressing carcinoma-associated fibroblasts synergizes with anti-PD-L1 immunotherapy in pancreatic cancer. Proc Natl Acad Sci U.S.A. (2013) 110. doi: 10.1073/pnas.1320318110 PMC3864274 24277834

[103] Carstens JL De Sampaio PC Yang D Barua S Wang H Rao A. Spatial computation of intratumoral T cells correlates with survival of patients with pancreatic cancer. Nat Commun. (2017) 8. doi: 10.1038/ncomms15095 PMC5414182 28447602

[104] Pillarisetty VG. The pancreatic cancer microenvironment: An immunologic battleground. Oncoimmunology. (2014) 3. doi: 10.4161/21624011.2014.950171 PMC4292569 25610740

[105] Kabacaoglu D Ciecielski KJ Ruess DA Algül H. Immune checkpoint inhibition for pancreatic ductal adenocarcinoma: Current limitations and future options. Front Immunol. (2018) 9. doi: 10.3389/fimmu.2018.01878 PMC6104627 30158932

[106] Morrison AH Byrne KT Vonderheide RH. Immunotherapy and prevention of pancreatic cancer. Trends Cancer. (2018) 4. doi: 10.1016/j.trecan.2018.04.001 PMC6028935 29860986

[107] Martinez-Bosch N Vinaixa J Navarro P. Immune evasion in pancreatic cancer: From mechanisms to therapy. Cancers. (2018) 10. doi: 10.3390/cancers10010006 PMC5789356 29301364

[108] Groeneveldt C Kinderman P Van Den Wollenberg DJM Van Den Oever RL Middelburg J Mustafa DAM. Preconditioning of the tumor microenvironment with oncolytic reovirus converts CD3-bispecific antibody treatment into effective immunotherapy. J Immunother Cancer. (2020) 8. doi: 10.1136/jitc-2020-001191 PMC7577070 33082167

[109] Zarezadeh Mehrabadi A Tat M Ghorbani Alvanegh A Roozbahani F Esmaeili Gouvarchin Ghaleh H. Revolutionizing cancer treatment: the power of bi- and tri-specific T-cell engagers in oncolytic virotherapy. Front Immunol. (2024) 15. doi: 10.3389/fimmu.2024.1343378 PMC10921556 38464532

[110] Pan H Liu J Deng W Xing J Li Q Wang Z. Site-specific PEGylation of an anti-CEA/CD3 bispecific antibody improves its antitumor efficacy. Int J Nanomedicine. (2018) 13. doi: 10.2147/IJN.S164542 PMC5985803 29881272

[111] Amgen. Pipeline (2024). Available online at: https://www.amgenpipeline.com/ (accessed May 03, 2024).

[112] Granger D Muczynski V Henne P Baccaro A Fernando D Shah M. NVG-222: A first-in-class autoregulating half-life extended ROR1xCD3 T cell engager heralding a new class of safer drugs. Blood. (2023) 142:2820–0. doi: 10.1182/blood-2023-189058

[113] Slaney CY Wang P Darcy PK Kershaw MH. CARs versus biTEs: A comparison between T cell–redirection strategies for cancer treatment. Cancer Discovery. (2018) 8. doi: 10.1158/2159-8290.CD-18-0297 30012854

[114] Klinger M Benjamin J Kischel R Stienen S Zugmaier G. Harnessing T cells to fight cancer with BiTE® antibody constructs - past developments and future directions. Immunol Rev. (2016) 270. doi: 10.1111/imr.2016.270.issue-1 26864113

[115] Martinelli G Boissel N Chevallier P Ottmann O Gökbuget N Topp MS. Complete hematologic and molecular response in adult patients with relapsed/refractory philadelphia chromosome-positive B-precursor acute lymphoblastic leukemia following treatment with blinatumomab: Results from a phase II, single-arm, multicenter study. J Clin Oncol. (2017) 35. doi: 10.1200/JCO.2016.69.3531 28355115

[116] Von Stackelberg A Locatelli F Zugmaier G Handgretinger R Trippett TM Rizzari C. Phase I/Phase II study of blinatumomab in pediatric patients with relapsed/refractory acute lymphoblastic leukemia. J Clin Oncol. (2016) 34. doi: 10.1200/JCO.2016.67.3301 27998223

[117] Pietzner K Woopen H Richter R Joens T Braicu EI Dimitrova D. Expression of epithelial cell adhesion molecule in paired tumor samples of patients with primary and recurrent serous ovarian cancer. Int J Gynecological Cancer. (2013) 23. doi: 10.1097/IGC.0b013e3182929056 23694980

[118] Ferrari F Bellone S Black J Schwab CL Lopez S Cocco E. Solitomab, an EpCAM/CD3 bispecific antibody construct (BiTE®), is highly active against primary uterine and ovarian carcinosarcoma cell lines *in vitro* . J Exp Clin Cancer Res. (2015) 34. doi: 10.1186/s13046-015-0241-7 PMC4609066 26474755

[119] Moek KL Fiedler WM von Einem JC Verheul HM Seufferlein T de Groot DJ. Phase I study of AMG 211/MEDI-565 administered as continuous intravenous infusion (cIV) for relapsed/refractory gastrointestinal (GI) adenocarcinoma. Ann Oncol. (2018) 29. doi: 10.1093/annonc/mdy279.414

[120] Hummel HD Kufer P Grüllich C Seggewiss-Bernhardt R Deschler-Baier B Chatterjee M. Pasotuxizumab, a BiTE®immune therapy for castration-resistant prostate cancer: Phase I, dose-escalation study findings. Immunotherapy. (2021) 13. doi: 10.2217/imt-2020-0256 33172323

[121] Amgen receives FDA priority review for tarlatamab BLA (2023). Available online at: https://www.pharmaceutical-technology.com/news/amgen-fda-priority-review-tarlatamab-bla/?cf-view (accessed January 18, 2024).

[122] FDA Grants Priority Review to Amgen’s Tarlatamab Application for Advanced Small Cell Lung Cancer (2023). Available online at: https://www.amgen.com/newsroom/press-releases/2023/12/fda-grants-priority-review-to-amgens-tarlatamab-application-for-advanced-small-cell-lung-cancer (accessed May 07, 2024).

[123] Matsuo K Taniguchi K Hamamoto H Inomata Y Komura K Tanaka T. Delta-like canonical Notch ligand 3 as a potential therapeutic target in Malignancies: A brief overview. Cancer Sci. (2021) 112:2984–92. doi: 10.1111/cas.v112.8 PMC8353941 34107132

[124] Food and Drug Administration. FDA grants accelerated approval to tarlatamab-dlle for extensive stage small cell lung cancer (2024). Available online at: https://www.fda.gov/drugs/resources-information-approved-drugs/fda-grants-accelerated-approval-tarlatamab-dlle-extensive-stage-small-cell-lung-cancer (accessed November 15, 2024).

[125] Chen J Xiao-Zhong G Qi XS. Clinical outcomes of specific immunotherapy in advanced pancreatic cancer: A systematic review and meta-analysis. J Immunol Res. (2017) 2017. doi: 10.1155/2017/8282391 PMC5318641 28265583

